# Development and validation of an occlusal cant index

**DOI:** 10.1186/s12903-022-02156-8

**Published:** 2022-04-15

**Authors:** Hessah A. Alhuwaish, Khalid A. Almoammar

**Affiliations:** Department of Pediatric Dentistry and Orthodontics, College of Dentistry, King Saud University, PO Box: 60169, Riyadh, 11454 Saudi Arabia

**Keywords:** Index, Occlusal cant, Occlusal tilt, Detection

## Abstract

**Background:**

Occlusal cant (OC) is a malocclusion trait lacking indexing or classification that describes the extent and severity of tilt in the occlusal plane. The aims of this study were to develop an occlusal cant index (OCI) based on the degree of OC detection among orthodontists and laypeople and to validate the newly developed OCI by a panel of experts using content validity.

**Methods:**

The ability to perceive OC was assessed in 134 participants (orthodontists = 67 and laypeople = 67). A frontal photograph of a model with an ideal smile with 0° of OC was obtained and manipulated to create various degrees of OC from 1–5° at the right and left sides. A set of 11 electronic photographs was displayed to the participants. The participants were asked to report whether they detected an OC in each photograph. The collected data was used as a baseline to develop an OCI. Then, a content validation of the OCI was performed using a questionnaire provided to a panel of experts comprising ten orthodontists.

**Results:**

The OCI was designed based on the threshold of OC detection. In both orthodontists and laypeople, the accuracy of OC detection increased as the amount of tilt increased. The threshold point of OC detection in orthodontists was at 2°, while in laypeople it was at 4°. There was a significant difference between orthodontists and laypeople in their ability to detect OC at 2–3° of tilt. The content validity index (CVI) showed excellent validity between the item-level CVI and the scale-level CVI of the OCI.

**Conclusion:**

The OCI was developed and implemented for diagnostic, communication, and research purposes. The index showed strong evidence supporting content validity.

## Background

An index is defined as “a numerical value describing the relative status of population on a gradual scale with definite upper and lower limits” [1]. Orthodontic indices are necessary to guide the practitioner with regard to communication, diagnosis, assessment of severity, treatment needs, and treatment outcomes [2, 3]. Shaw et al. [2] divided orthodontic indices into five categories: (1) diagnostic indices, such as Angle’s classification [4], (2) epidemiological indices, such as Little’s irregularity index [5], (3) indices regarding orthodontic treatment needs, such as the Index of Orthodontic Treatment Need [6], (4) indices regarding orthodontic treatment outcomes, such as Peer Assessment Rating Index [7], and (5) indices conveying treatment complexity, such as Index of Complexity, Outcome, and Need [8].

Occlusal plane (OP) is a cornerstone element in smile analysis, and occlusal canting is a malocclusion trait of esthetic concern that must be evaluated carefully during orthodontic assessment [9]. Any vertical alteration or rotation of the OP in the transverse plane of one side over the other is considered an occlusal cant (OC) [10]. OC could be skeletal or dental in origin and may present with or without facial asymmetry; however, several studies have reported a high association between OC and facial asymmetry [11–14]. A high prevalence—up to 41%—of OCs in patients with Class III malocclusions has been observed [12]. OC is a malocclusion trait that lacks indexing or classification [10]. The development and utilization of a common index for OC will facilitate an international language for clinical communication among practitioners, as well as accurate diagnoses of the site and the amount of OC. Such an index will also open up new areas of research in clinical or epidemiological studies. Designing a simple index for OC is an important first step to facilitate the future development of international guidelines for the assessment and treatment of OC.

The development of a diagnostic index for OC severity and categorization was designed in this study to be based on OC detection among orthodontists and laypeople. Previous studies have investigated the perception of OC, they found that orthodontists and laypeople are capable of detecting OCs to varying degrees [10, 11, 15]. They have demonstrated that OC detection ability is commonly observed at a range of 2° to 4°[15, 16]. Orthodontists, given their professional background, are more accurate in identifying OC at lower rates [15]. Ker et al. found that laypeople can readily detect OC at 4 ° and 6 ° [17]. This variation in detection capacity is most likely attributed to differences in professional expertise, knowledge, and professional environment [18]. Orthodontists, according to Kokich et al., consider OC as the most obvious discrepancy in smile characteristics, whereas laypeople value crown angulation as an obvious feature [19].

The OC detection ability among orthodontists and laypeople would be the base to design a new index for OC. One of the main requirements for new index is to be valid, which is defined as “the degree of which the method measures what it is meant to measure” [20]. Content validation is the initial step towards full validation following the development of an index or scale and is considered to be an expert’s subjective judgment on the degree of relevance and clarity a scale’s content. Furthermore, it provides the required preliminary evidence for testing a newly devised index and highlights the need for any modifications prior to the next level of validation: objective validity [21–24].

OC has rarely been covered and evaluated in the literature. It has clinical implications for function and aesthetic [11]. To the best of our knowledge, there is no established index or classification describing the extent and severity of tilt in the OP, hence, the novelty of this study lies in proposing an index of OC that has never been proposed in the past. The aims of this study were: (1) to develop an occlusal cant index (OCI) based on the degree of OC detection among orthodontists and laypeople and (2) to validate the newly developed OCI by a panel of experts using content validity. The null hypothesis that there was no difference among the experts in validating the newly developed OCI.

## Methods

The OCI development and validation process underwent three processes: the OC detection, designing OCI, and the content validation of OCI.

The development of the OCI was based on the measurement of OC detection among orthodontists and laypeople based on the evaluation of various degrees of OC; the data was then used as a baseline to develop the OCI.

**Fig. 1:**
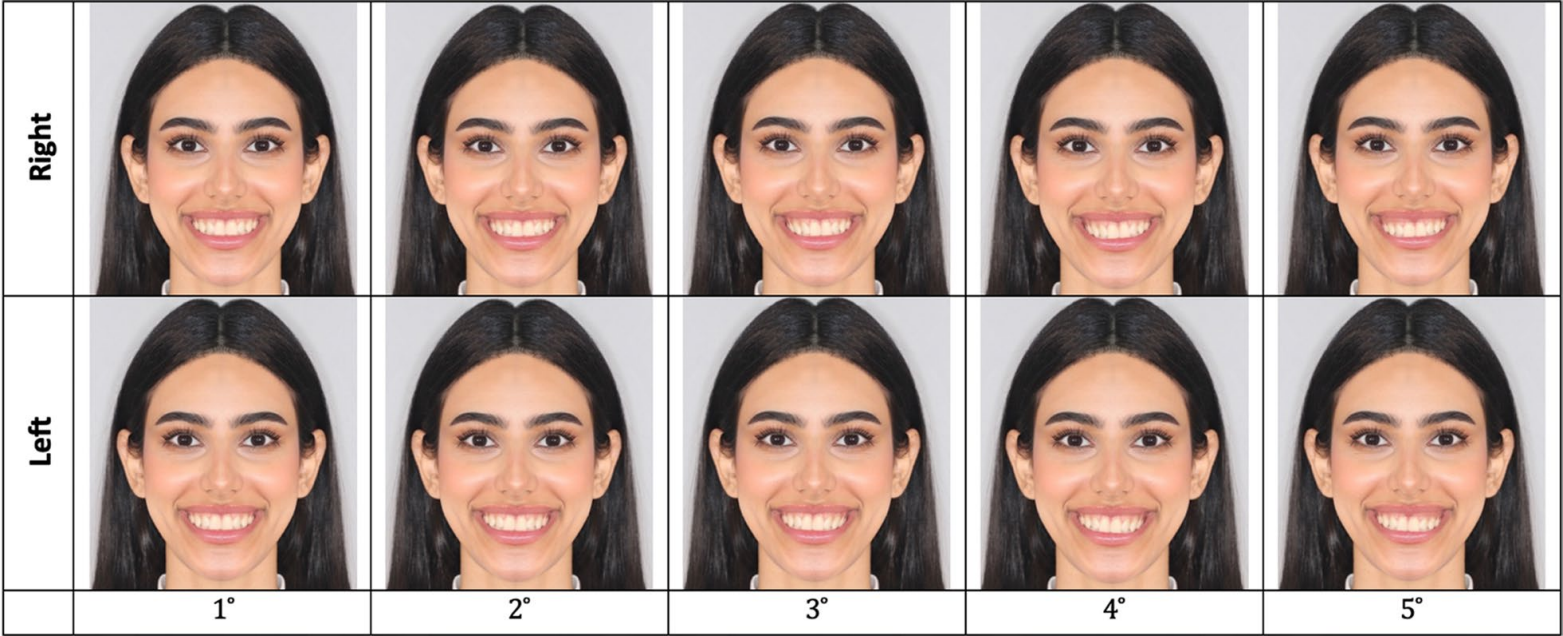
Occlusal plane manipulation: **a** right-sided OC 1°–5°; **b** left-sided OC 1°–5°

To identify the OC detection threshold, photographs were obtained from a patient selected from the orthodontic clinic of the Dental University Hospital at King Saud University based on the following criteria: adult, absence of any facial asymmetry, no history of extraction, absence of any external distractor—such as eyeglasses—that may influence the evaluation, and the presence of ideal esthetic smile characteristics [25]. Two photographs were taken using a digital camera (Cannon Digital, A610, Tokyo, Japan): one extraoral photograph of a natural head position with a spontaneous smile, and one frontal intra-oral photograph with the camera placed at the OP level. The photographs obtained from the model were manipulated to create different degrees of OC using Photoshop software (Adobe Photoshop 9.0, CA, USA). For accurate manipulation, the interpupillary line in the extraoral photograph was used as a reference to digitally rotate the OP in the frontal intraoral photograph. One photograph with 0° OC was considered the original photograph. Then, through the manipulation process, the OP in the original photograph was rotated in 1° increments from 1° to 5° in a clockwise direction on the right side only. The five manipulated photographs were then flipped horizontally to create the left-sided OC (Fig. 1). For standardization purposes, the image was flipped horizontally; hence, only one side of the face would have to be manipulated to produce the desired degrees of occlusal tilt. The patient signed a consent form allowing for the use of her photographs in all desired manipulations for this study.

A sample size estimation based on a power of 0.9 at a p-value of 0.05 confirmed that the required number of participants to be enrolled was 134. Accordingly, 134 individuals participated in the study, 67 of which were orthodontists from the Dental University Hospital at King Saud University. Each of these orthodontists had a minimum of three years of experience. The remaining 67 participants were laypeople randomly selected from among nonmedical employees at the Dental University Hospital at King Saud University (Table 1). Written informed consent was obtained from all the participants enrolled in the study. The questionnaire was prepared electronically using the survey software Alchemer (Alchemer, Boulder, CO, USA) and displayed to the participants on a tablet device (Apple iPad Pro 11, Apple Inc., Cupertino, CA, USA). The questionnaire was designed to commence with items that collected participants’ demographic data, including sex and profession. These items were followed by a set of randomly arranged photographs from Fig. 1 showing different degrees of OC. To ensure that the manipulated photographs were viewed under optimal standardization conditions, the tablet device was set to a brightness of 50% and a contrast of 100%. The participants were asked to report whether they detected an OC in each photograph in less than 40 S. For inter and intra-examiner reliability assessments, 20 of the participants (10 orthodontists and 10 laypeople) were randomly selected to repeat the questionnaire after two weeks.

**Table 1:** Sample distribution of individuals recruited for OC detection

Participants	N	Male	Female
Orthodontists	67	31	36
Laypeople	67	33	34
Total	134	57	77

Later, the data collected from occlusal cant detection were used as a baseline to develop an OCI. The average of the starting points, measured in degrees, of OC detection by the orthodontists and laypersons served as the boundaries or cut-off points among index grades. The index consists of four grades: grade 0 refers to the absence of an OC and the OP is parallel to the true horizontal plane; grade I denotes mild OC that could not be detected by either set of evaluators (orthodontists and laypersons); grade II indicates a range of OC degrees identified only by the orthodontists; and grade III represents severe OC cases wherein the degrees of OC are detected by both the orthodontists and the laypersons. For a comprehensive description of the OC cases in the index, each grade is accompanied by the site (right or left side), with the OP tilted downward (Table 2).

**Table 2:** The proposed description of the OCI grades

Grade	Side	Description
Grade 0		No OC is present (the OP is parallel to the true horizontal plane)
Grade I	Right	The OP is tilted down on the right side, and the OC is NOT detected by either the orthodontists or the laypersons
	Left	The OP is tilted down on the left side, and the OC is NOT detected by either the orthodontists or the laypersons
Grade II	Right	The OP is tilted down on the right side, and the OC is detected by the orthodontists only
	Left	The OP is tilted down on the left side, and the OC is detected by the orthodontists only
Grade III	Right	The OP is tilted down on the right side, and the OC is detected by the orthodontists and the laypersons
	Left	The OP is tilted down on the left side, and the OC is detected by the orthodontists and the laypersons

In order to validate the newly developed index, ten orthodontists from the Dental University Hospital at King Saud University with more than 10 years of experience were invited to participate in the validation process. Written informed consent was obtained from all evaluators enrolled in this study. The recommended range of experts for content validation studies is 5–10 [15–18]. The questionnaire was prepared electronically using the Alchemer survey software (Alchemer, Boulder, CO, USA) and displayed to the experts on a tablet device (Apple iPad Pro 11, Apple Inc.). The questionnaire commenced with the OCI table, which was presented and explained to the experts. Next, a set of the items to be assessed were presented as questions. The evaluators were then asked to rate each item based on relevance and clarity on a four-point scale (Table 3).

**Table 3:** Items and assessment criteria of the content validity questionnaire

**Diagnosis of the OC**				
1a. Is the OCI relevant to the diagnosis of the OC?	1 Not relevant	2 Relevant but needs major revisions	3 Relevant but needs minor revisions	4 Very relevant
1b. Is the OCI clear to the diagnosis of the OC?	1 Not clear	2 Clear but needs major revisions	3 Clear but needs minor revisions	4 Very Clear
**Side of the OC**				
2a. Is the OCI relevant with respect to detecting the side of the OC?	1 Not relevant	2 Relevant but needs major revisions	3 Relevant but needs minor revisions	4 Very relevant
2b. Is the OCI clear to detecting the side of the OC?	1 Not clear	2 Clear but needs major revisions	3 Clear but needs minor revisions	4 Very clear
**Cut-off points of the scoring system**				
3a. Are the cut-off points of the scoring systems being relevant?	1 Not relevant	2 Relevant but needs major revisions	3 Relevant but needs minor revisions	4 Very relevant
3b. Are the cut-off points of the scoring systems clear?	1 Not clear	2 Clear but needs major revisions	3 Clear but needs minor revisions	4 Very clear
**Communication**				
4a. Is the OCI relevant with respect to communication among practitioners and researchers?	1 Not relevant	2 Relevant but needs major revisions	3 Relevant but needs minor revisions	4 Very relevant
4b. Is the OCI relevant with respect to communication among practitioners and researchers?	1 Not clear	2 Clear but needs major revisions	3 Clear but needs minor revisions	4 Very clear
**Foundation for future modifications**				
5a. Is the OCI as a foundation index relevant for any applicable future modification?	1 Not relevant	2 Relevant but needs major revisions	3 Relevant but needs minor revisions	4 Very relevant
5b. Is the OCI as a foundation index clear for any applicable future modification?	1 Not clear	2 Clear but needs major revisions	3 Clear but needs minor revisions	4 Very clear

### Statistical analysis

All data were analyzed using the Statistical Package for the Social Sciences (SPSS) software version 26.0 (IBM Inc., Chicago, IL, USA). Descriptive statistics were used to describe all variables.

A significant difference in OC detection between laypeople and experts was calculated (∝ = 0.05) using the chi-squared test. To evaluate the inter- and intra-examiner reliability in OC detection among orthodontists and laypeople, kappa statistics were used. For the assessment of the content validity of the OCI, the content validity index (CVI) was used, including both the item-level CVI (I-CVI), which measures the proportion of experts who provided a rating of 3 or 4 to each item, and the scale-level CVI based on average (S-CVI/Ave) which reflects the average of I-CVI scores for all items on the OCI. The OCI is considered to have excellent content validity if I-CVI was equal to or more than 0.78 and S-CVI/Ave was equal to or more than 0.9; otherwise, a revision based on the experts’ opinions was deemed necessary. In addition, a modified kappa index (κ*) of inter-rater agreement is an important supplement to CVI. It was computed to provide information about the degree of agreement by eliminating any random elements.

## Results

### OC detection

The inter- and intra-examiner reliabilities among laypeople were (0.83) and (0.86) while among orthodontists were (0.92) and (0.89) respectively, which indicate high kappa values In both groups, there were no significant differences in OC detection between sexes; accordingly, the data were pooled.

Orthodontists were able to detect the OC at all degrees except for 1° on both sides (Table 4). On the other hand, the ability to detect OC was significantly reduced among laypeople, as they were only able to detect OC at 4° and 5° on both sides (Table 4).

**Table 4:** OC detection at varying degrees of OC among orthodontists and laypeople

	Orthodontists N = 67			Laypeople N = 67		
Photo	Count (%)	Chi-Square	*P*	Count (%)	Chi-Square	*P*^c^
5° L^a^	65 (97)	59.239	0.00***	60 (89.55)	41.925	0.00***
4° L	60 (89.60)	41.925	0.00***	53 (79.10)	22.701	0.00***
3° L	56 (83.60)	30.224	0.00***	24 (35.80)	5.388	0.02*
2° L	46 (68.70)	9.328	0.002**	21 (31.30)	9.328	0.002**
1° L	7 (10.40)	41.925	0.00***	7 (10.40)	41.925	0.00***
0°	2 (3)	59.239	0.00***	4 (6)	51.955	0.00***
1° R^b^	9 (13.40)	35.836	0.00***	8 (11.90)	38.821	0.00***
2° R	55 (82.10)	27.597	0.00***	23 (34.30)	6.582	0.01**
3° R	60 (89.60)	41.925	0.00***	24 (35.80)	5.388	0.02*
4° R	60 (89.60)	41.925	0.00***	56 (83.60)	30.224	0.00***
5° R	65 (97)	59.239	0.00***	64 (95.50)	55.537	0.00***

^a^*L* Left; ^b^*R* Right, ^c^*P-*value*:* * = *P* ≤ 0.05, ** = *P* ≤ 0.01, *** = *P* ≤ 0.001

A comparison of OC detection between orthodontists and laypeople in Table 5 shows that orthodontists had an increased ability to detect OC compared to laypeople. There was a statistically significant difference (p < 0.05) between the groups at 2° and 3° on both sides; the orthodontists were found to be more able to detect OC. Accordingly, the OC detection thresholds among orthodontists and laypeople were measured at 2° and 4°, respectively.

**Table 5:** Comparison between orthodontists and laypeople in OC detection ability

Degree	Count (%)		Chi-Square	*P*^c^
	Orthodontists N = 67	Laypeople N = 67		
5° L^a^	65 (97.00)	60 (89.55)	2.978	0.084
4° L	60 (89.60)	53 (79.10)	2.767	0.096
3° L	56 (83.60)	24 (35.80)	31.763	0.00***
2° L	46 (68.70)	21 (31.30)	18.657	0.00***
1° L	7 (10.40)	7 (10.40)	0	1
0°	2 (3.00)	4 (6.00)	0.698	0.403
1° R^b^	9 (13.40)	8 (11.90)	0.067	0.795
2° R	55 (82.10)	23 (34.30)	31.414	0.00***
3° R	60 (89.60)	24 (35.80)	41.349	0.00***
4° R	60 (89.60)	56 (83.60)	1.027	0.311
5° R	65 (97.00)	64 (95.50)	0.208	0.649

^a^*L* Left; ^b^*R* Right, ^c^*P-*value*:* * = *P* ≤ 0.05, ** = *P* ≤ 0.01, *** = *P* ≤ 0.001

### OCI development and validation

Data collected from the OC detection were used as a baseline to develop the OCI and define the degrees of OC in each grade (Table 6).

**Table 6:** Occlusal cant index (OCI)

Grades	Degree	Side	Descriptions
Grade 0	0°		No OC is present (the OP is parallel to the true horizontal plane)
Grade I	1°	Right	The OP is tilted down on the right side by 1°
	1°	Left	The OP is tilted down on the left side by 1°
Grade II	2°–3°	Right	The OP is tilted down on the right side by 2–3°
	2°–3°	Left	The OP is tilted down on the left side by 2–3°
Grade III	≥ 4°	Right	The OP is tilted down on the right side by ≥ 4°
	≥ 4°	Left	The OP is tilted down on the left side by ≥ 4°

Ten experts scored five items regarding two attributes (relevance and clarity). In the item-level CVI, the relevance and clarity of the OCI were measured at equal or more than 0.78 I-CVI and more than 0.74 κ*; these results are interpreted as showing excellent content validity. The CVI for the entire OCI was calculated in terms of relevance and clarity by scale-level CVI based on the average S-CVI/Ave and scored 0.94 and 0.92, respectively, where S-CVI/Ave is equal or more than 0.9 is considered the goal value of for high content validity (Table 7).

**Table 7:** Content validity of the OCI

		Relevance					Clarity				
Items	Number of experts	Number of ratings of 3 or 4	I-CVI^a^	P_C_^b^	κ*^c^	Evaluation^d^	Number of ratings of 3 or 4	I-CVI^a^	P_C_^b^	κ*^c^	Evaluation^d^
1	10	9	0.90	0.009	0.90	***	9	0.90	0.009	0.90	***
2	10	10	1.00	0.001	1.00	***	10	1.00	0.001	1.00	***
3	10	10	1.00	0.001	1.00	***	9	0.90	0.009	0.90	***
4	10	9	0.90	0.009	0.90	***	9	0.90	0.009	0.90	***
5	10	10	1.00	0.001	1.00	***	10	1.00	0.001	1.00	***
S-CVI/Ave^e^		0.96					0.94				

^a^I-CVI (item-level content validity index) = number of experts providing a rating of 3 or 4 / number of experts

^b^Pc (probability of chance occurrence) = [N/A * (N - A)] × 0.5^ N^, N = number of experts; A = number of experts providing a rating of 3 or 4

^c^κ*(modified kappa) = (I-CVI-Pc)(1-Pc)

^d^Evaluation criteria for the level of content validity: relationship between I-CVI and k*; excellent validity = I-CVI ≥ 0.78 and k* > 0.74 (****); good validity I-CVI < 0.78 and ≥ 0.60 and k* ≤ 0.74 (***); fair validity I-CVI < 0.6 and ≥ 0.40 and k* ≤ 0.59 (**); and poor validity I-CVI < 0.4 and κ* < 0.40 (*)

^e^S−CVI/Ave (scale−level content validity index based on the average agreement among experts) = sum of the I−CVI / number of items

## Discussion

OC is a malocclusion trait currently lacking indexing or classification. The purpose of this study was to develop a newly proposed index to classify OC. The classification designed in this study was based on the detection thresholds of OC among orthodontists and laypeople. The current literature lacks a common consensus to categorize the wide range of OC. Classifications and indices are essential in providing a basis for a rational, coherent, and systematic framework for categorizing and analyzing a disease or trait [26]. The design of an index for OC will facilitate clinical assessment and diagnosis, as well as form the basis for epidemiological and research purposes regarding OC.

In the process of designing a new index involved ascertaining the ability of orthodontists’ and laypeople to detect OC, which was found to have increased for both observed groups as the amount of tilt increased. We found that there was a significant difference between orthodontists and laypeople in their ability to accurately detect OC; orthodontists detected all degrees of OC except for cases measured at 1°, while laypeople were able to perceive OC significantly at 4° and 5°. According to these findings, the OC thresholds were determined for each category. Grade I was defined as 1° OC, which is an amount of OC undetectable by orthodontists or laypeople. The grade II range of 2°–3° reflects the category of OC detected by orthodontists only. Grade III is measured at 4° and detectable by both orthodontists and laypeople.

The findings of this current paper are consistent with a US study that found that laypeople detected OC at 4 ° [19]. Ker et al. [17] also found that laypeople were only capable of detecting OC at 4°, while one-third of their sample accepted the tilt at 6°. Recent work by Shiyan et al. [15] demonstrated that orthodontists were more precise in detecting OC than laypeople. This variation in detection capacity is most likely attributable to differences in professional expertise, knowledge, and professional environment [18]. Orthodontists, according to Kokich et al. [19], consider OC to be the most obvious discrepancy in smile characteristics. This may also explain our finding that the percentage of orthodontists who perceived OC was higher than that of laypeople in all 11 variations of OC presented.

The study participants included orthodontists with a minimum of three years of experience and laypeople with no medical or dental background to influence their decisions. The inclusion of laypeople in the study served to represent social opinion. It is well documented that laypeople have their own criteria for what constitutes an ideal smile [15, 19, 27]. As such, laypeople’s diminished ability to detect irregularities or abnormalities in comparison to dental professionals may serve as a deterrent to the recommendation or preparation of unnecessary treatment plans and complex approaches that may, in reality, be deemed irrelevant in an esthetic context [17]. In addition, the selected photograph represented a posed smile for standardization, and the image was flipped horizontally during manipulation, as described earlier. In this manner, human errors in manipulation and any asymmetry between the right and left sides of the model were eliminated.

As previously mentioned, the validation process is a cornerstone in the development of a new index. This ensures both usability for diagnostic purposes and the future development of international guidelines for the assessment and treatment of OC. A content validity evaluation was performed to provide preliminary evidence for testing the newly devised index and highlighting the possibility of any modifications. The null hypothesis that there was no difference among the experts in validating the newly developed OCI is accepted. The proposed OCI had an excellent validity (S-CVI/Ave ≥ 0.9). All studied items in relation to the relevance and clarity of OCI were measured at ≥ 0.78 I-CVI and > 0.74 κ*, representing high content validity. For each item, the relevance and clarity of the content were evaluated. The experts differentiated and identified differences between the relevance and clarity of the content. They were clearly satisfied with the wording of the items in the index. A modified kappa index was utilized to test for the chance of agreement, which showed excellent agreement across the items. It is well documented that the CVI and kappa agreement results reflect a precise process for content validation evaluation [28]. In evaluating the content of the index, experts with a minimum of 10 years of experience in the field of orthodontics were invited to evaluate and modify the scale, if required.

This proposed index will serve as a diagnostic tool for OC in the clinical examination process. It indicates the extent and severity of this occlusal trait, as well as highlights the location or side of tilt in the OP. It includes grades for OCs, measured in degrees, and describes OC occurring on the right and left sides. This comprehensiveness will aid in communication among professionals. This index will provide a valid clinical tool for clinical diagnosis and facilitate communication among professionals. It also has applications in the education and epidemiological spectrums.

Additionally, this index is straightforward and simple to use. It is easily incorporated into routine clinical practice since it requires little or no time to set up. The index has the advantage of being amenable to future modifications. Adjustments may be made to further extend the categorization to include items concerned with the origin of OC, whether the tilt is caused skeletally or as a result of dental discrepancies.

This study has several limitations. For example, in the detection phase, a single smile image of a female human was used, which has previously been reported to influence smile attractiveness [29]. Photographs of female models tend to be rated at lower scores for smile beauty when compared to photos of male models [29]. Another limitation was the use of a posed smile only, instead of different smile heights. According to Shiyan et al. [15], different levels of smile height may affect the perception of OC among experts and laypeople alike. Excessive exposure of the anterior gingiva is also a confounding factor that may affect anterior smile aesthetics and make transverse anterior cants less acceptable in high-smile line groups [15]. The index’s classification is confined to two elements of OC: the amount and location of the discrepancy. In this study, only a content validation assessment was employed, which was crucial in identifying the validity of the content measures. This is the first step required in the route to complete validation. Hence, future studies should test the criterion validation of the instrument to evaluate the validity of this diagnostic tool.

## Conclusion

The OCI was developed to be implemented for diagnostic, communication, and research purposes. The index showed strong evidence supporting content validity.

## Data Availability

The datasets used and/or analyzed during the current study are available from the corresponding author on reasonable request.

## Abbreviations

OP: Occlusal plane
OC: Occlusal cant
OCI: Occlusal cant index
CVI: Content validity index
I-CVI: Item-level content validity index
S-CVI: Scale-level content validity index
